# Association of Serum Cystatin C Level With Carotid Arterial Wall Elastic Resistance as a Potential Marker for Detection of Early Stage Atherosclerosis

**DOI:** 10.7759/cureus.38543

**Published:** 2023-05-04

**Authors:** Vishnu Chander S, Sarumathy S, Nanda Kumar R, Meenakshi Sundari S N, Anuba P A

**Affiliations:** 1 Department of General Medicine, SRM Institute of Science and Technology, SRM Medical College Hospital and Research Centre, Faculty of Medicine and Health Sciences, Kattankulathur, IND; 2 Department of Pharmacy Practice, SRM Institute of Science and Technology, SRM College of Pharmacy, Faculty of Medicine and Health Sciences, Kattankulathur, IND

**Keywords:** cardiovascular diseases, cysteine proteinase inhibitor, cystatin-c, carotid wall elastic modulus, atherosclerosis

## Abstract

Background: Early diagnosis of atherosclerosis is exigent in patients with known cardiovascular disease (CVD) risk factors. During the initial phases of atherosclerosis, appearance of plaques can be detected by the ultrasonic phased tracking method which measures the arterial wall elasticity. However, reliable and easily available biochemical markers are not evaluated in the diagnosis of early-stage atherosclerosis. So the current study was carried out to assess the serum cystatin C level as an atherosclerotic marker, by evaluating its association with carotid arterial elastic modulus using the phased tracking method.

Materials and methods: A cross-sectional study was conducted on 115 patients having risk factors for atherosclerosis but not meeting carotid intima-media thickness (IMT) criteria. The early-stage atherosclerosis was detected by using the ultrasonic phased tracking method and the patients were divided based on low and high carotid elastic modulus. Serum levels of cystatin-C were measured in association with IMT, and elastic modulus was calculated using a novel method. This study also put forth the evaluation of the sensitivity and specificity of cystatin C for early diagnosis of atherosclerosis.

Results: Cystatin C was strongly related to carotid elasticity (r=0.650). Based on multi-linear regression analysis, cystatin C showed significant association with carotid elasticity (β=0.509; p<0.001). It also displayed significant positive association with high carotid elastic modulus (β=0.511; p=0.02). Cystatin C showed a sensitivity of 85% in the prediction of high carotid elastic modulus.

Conclusion: For patients who are at risk to evolve atherosclerosis but are not evident with arterial plaques, cystatin C exhibits a significant association with carotid wall elastic modulus, which eases the detection of atherosclerosis. Thus, cystatin C is a potential biochemical marker for early diagnosis of atherosclerosis.

## Introduction

Globally, atherosclerotic cardiovascular disease (ASCVD) is one of the major causes of mortality and imposes a significant economic burden [1]. A recent meta-analysis reported that worldwide around 816 million people are affected with carotid plaque and 58 million with carotid stenosis [2]. Atherosclerosis remains the major cause of cardiovascular disease (CVD). There are several non-invasive tests used for evaluating atherosclerosis in clinical practice. Mounting clinical studies shows that metabolic syndrome, alcohol and smoking are major risk factors for atherosclerosis [3-4] and aggravate CVD morbidity and mortality in the general population [5-6].

Non-invasive measurement of carotid atherosclerosis such as carotid intima-media thickness (CIMT) and carotid plaque (CP) are evaluated using B-mode ultrasonography which remains reliable marker for early detection of systemic atherosclerosis and cardiovascular disease [7]. Previous report shows that intima-media thickness (IMT) is a reliable marker of systemic subclinical atherosclerosis and an effective predictor of incident myocardial infarction and ischemic stroke. Recently, for detecting early-stage atherosclerosis, before analyzing substantial morphological changes in carotid IMT, a non-invasive ultrasonic phased tracking method is developed which measures multiple site deformities in arterial walls during a single heartbeat [8].

Earlier studies with ultrasonic phased tracking, which measures the arterial wall elasticity, displayed a significant association with various CVD risk factors such as diabetes, dyslipidemia, hypertension, smoking and alcohol. In contrast, IMT value less than 1.1mm, pulse wave velocity and plaque score are not significantly associated with these risk factors and thus these patients are not classified as having atherosclerosis according to IMT criteria [9]. In addition, carotid artery wall elasticity is associated with visceral fat mass in obese patients and also in patients with CVD risk factors [10]. Furthermore, arterial wall elasticity is significantly elevated in heavy smokers compared to nonsmokers [11] and in patients with metabolic syndrome. Major risk factors for metabolic syndrome include obesity, elevated blood pressure and abnormal lipid levels [12]. The above findings illustrate the significance of measuring arterial wall elasticity for early detection of atherosclerosis before assessing the substantial changes in IMT values. However, there is a need for reliable serum biochemical markers for the detection of early-stage atherosclerosis which has good association with arterial wall elasticity.

Cystatin C, a 13-kD endogenous cysteine proteinase inhibitor produced by all nucleated cells at a constant rate, is found in all tissues and body fluids. Cystatin C plays an important role in the atherosclerotic process, i.e., inhibiting the cathepsin-dependent proteolytic activity in the vascular wall. The remodeling of the extracellular matrix (ECM) in the vascular wall is an important feature of atherosclerosis pathogenesis. The imbalance between cathepsin and cystatin C in vascular local sites may result in increased degradation of ECM, leading to the development of an atherosclerotic plaque. Indeed, human pathological studies have shown increased cathepsin and decreased cystatin C expression in atherosclerotic lesions [13]. Further, elevated levels of cystatin C are significantly associated with subclinical atherosclerosis [14] and plaque morphology [15] among patients without chronic kidney disease. In addition, irrespective of estimated glomerular filtration rate (eGFR), cystatin C was found to be associated with the presence of CVD in patients with mild renal impairment [16]. Several population‐based studies have shown that elevated plasma cystatin C levels are associated with CVD events [17]. Patients with coronary heart disease with elevated cystatin C are also at higher risk after adjustment for traditional risk factors [18]. A study showed that elevated cystatin C level in the general population was associated with the presence of classical cardiovascular risk factors such as diabetes, hypertension and chronic renal disease, along with higher levels of C-reactive protein, homocysteine and fibrinogen [19].

Against this backdrop, the present study was done to evaluate the association between serum cystatin C and arterial wall elasticity, measured by using a phased tracking method in patients who are at risk for atherosclerosis. Further, the sensitivity and specificity of cystatin C in early detection of atherosclerosis were also studied.

## Materials and methods

This was a cross-sectional study conducted among 115 healthy individuals with at least one established risk factor for atherosclerosis such as diabetes mellitus, systemic hypertension, or sedentary lifestyle. This study was approved by the Institutional Ethics Committee of SRM Medical College Hospital and Research Centre and attained ethics clearance number 1700/IEC/2019. This approved cross-sectional study was carried out in a tertiary care hospital in India for a period of 18 months. Informed written consent was obtained from all the study participants, and only those participants willing to sign the informed consent were included in the study.

Inclusion and exclusion criteria

Male and female subjects of age more than 30 years and below 70 years and subjects with CIMT ≤ 0.8mm were included. Individuals with any of the following cardiovascular risk factors such as hyperlipidemia, Type II diabetes mellitus (T2DM), systemic hypertension, sedentary lifestyle, obesity, smoking (smoking tobacco in any form for a minimum of three months with at least one cigarette per day) or alcohol (consumable ethanol usage in any form for minimum three months and at least 45ml per day) were included in the study. For assessing patients for risk factors following criteria under each category has to be evaluated for the inclusion of the patient: for T2DM (i) previously diagnosed as T2DM and on medication (ii) elevated fasting blood sugar (FBS) ≥126mg/dl, post prandial blood sugar (PPBS) ≥200mg/dl and glycated hemoglobin (HbA1C) ≥6.5; for hypertension (i) systolic blood pressure 140-159mm/Hg and diastolic blood pressure 90-99mm/Hg; for hyperlipidemia (i) low-density lipoprotein (LDL) ≥130mg/dl (ii) high-density lipoprotein (HDL) <40mg/dl (iii) total cholesterol >200mg/dl. According to National Cholesterol Education Program's (NCEP) Adult Treatment Panel III guidelines, the defining level of HDL cholesterol in men is <40mg/dl and for women it is measured to be <50 mg/dl. Patients with pregnancy, thyroid disorder, tumor and severe inflammation, with atrial fibrillation, CIMT ≥0.8, chronic kidney disease and rheumatology disorders were excluded from the study.

Measurement of elastic modulus in longitudinal direction (εθ)

Carotid artery elasticity in the carotid artery was evaluated using the phased tracking method. The arteries were scanned with ultrasonic diagnostic equipment Philips A30 and a linear type probe with a high-frequency range of 9MHz in the supine position. The IMC will be longitudinally scanned using B-mode ultrasonography at two locations on the far wall of the common carotid artery, 1cm and 3cm proximal to the bifurcation [9]. Multiple sites from the luminal surface to the adventitia along each beam were programmed during the quadrature demodulation of the IMC signals throughout eight heartbeats, with one heartbeat having a precise image. The displacement of each point during one heartbeat was obtained by applying the phased tracking method. The formulae used were as follows:

Er = ∆P⁄ε

ε=Δh⁄h_0_ 

Δh is the maximum decrease in thickness of the arterial wall during one heartbeat. ∆P is the pulse pressure, difference in systolic blood pressure and diastolic blood pressure.

εθ=(3⁄8)×((2r_0_)/h_0_ +1)×Er

r_0_ initial thickness of vessel at end diastole

h_0_ initial radius of vessel at end diastole

Measurement of carotid artery intima media thickness (CIMT)

This was measured at a point on the far wall of the common carotid artery, in a 1cm segment proximal to the bifurcation. Mean value of bilateral measurement was used for analysis. Cystatin C was assayed using a colloidal gold immunoassay.

Statistical analysis

Association between quantitative variables was analyzed by Pearson’s correlation and multilinear regression. The utility of cystatin C (mg/L) in predicting E theta was assessed by receiver operative curve (ROC) analysis. Area under the ROC curve along with 95% CI and P value were presented. P value < 0.05 was considered to be statistically significant. Statistical analysis was calculated using Statistical Package for the Social Sciences (SPSS; IBM Corp., Armonk, NY, USA) software version 2. 

## Results

In this study, 115 subjects were enrolled for the final analysis of the study. The overall demographics and clinical characteristics of the patients are listed in Table 1. Study participants were categorized by carotid elastic modulus (kPa) values as high elastic modulus (n=63) and low elastic modulus (n=52) groups. The comparison of demographics and clinical characteristics of the patients between high (n=63) and low (n=52) elastic modulus were also illustrated in Table 1.

**Table 1:** Patient demographics and clinical characteristics in low and high carotid elasticity groups. * denotes significant p <0.05. SBP: systolic blood pressure; DBP: diastolic blood pressure; BMI: body mass index; BUN: blood urea nitrogen; FBS: fasting blood sugar; PPBS: post prandial blood sugar; HbA1c %: glycated hemoglobin; LDL: low density lipoprotein; HDL: high density lipoprotein; VLDL: very low density lipoprotein; TGL: triglycerides; IMT: intima-media thickness

Parameters	All subjects (n=115)	Low Eθ (n=63)	High Eθ (n=52)	P value
Age (years)	47.08 ± 6.26	46.83 ± 6.07	47.38 ± 6.52	0.63
Gender				
Male	66 (57.4%)	27 (40.91%)	39 (59.09%)	0.001*
Female	49 (42.6%)	36 (73.47%)	13 (26.53%)	
SBP (mm Hg)	127.44 ± 8.25	126.05 ± 8.30	129.12 ± 7.95	0.04*
DBP (mm Hg)	81.97 ± 5.9	81.13 ± 6.22	82.98 ± 5.37	0.08
BMI (kg/m^2^)	26.56 ± 2.82	26.46 ± 2.81	26.70 ± 2.86	0.65
Creatinine (mg/dl)	0.75 ± 0.18	0.74 ± 0.18	0.76 ± 0 .18	0.61
Urea (mg/dl)	31.55 ± 5.07	31.48 ± 4.74	31.63 ± 5.49	0.87
BUN (mg/dl)	14.72 ± 2.37	14.69 ± 2.21	14.76 ± 2.56	0.87
FBS (mg/dl)	105.37 ± 21.78	101.87 ± 19.82	109.60 ± 23.43	0.06
PPBS (mg/dl)	146.58 ± 20.65	144.71 ± 19.99	148.85 ± 21.39	0.29
HbA1c %	6.62 ± 0.78	6.56 ± 0.76	6.69 ± 0.80	0.37
Total Cholesterol (mg/dl)	193.77 ± 37.38	189.09 ± 40.21	199.44 ± 33.14	0.13
LDL (mg/dl)	134.31 ± 33.56	127.87 ± 34.64	142.12 ± 30.73	0.02*
HDL (mg/dl)	35.58 ± 7.29	36.63 ± 8.02	34.30 ± 6.13	0.08
VLDL (mg/dl)	23.88 ± 4.89	24.59 ± 5.38	23.02 ± 4.12	0.08
TGL (mg/dl)	147.67 ± 15.48	144.89 ± 14.69	151.03 ± 15.89	0.03*
IMT (mm)	0.72 ± 0.04	0.72 ± 0.04	0.72 ± 0.04	0.55
Cystatin C (mg/L)	0.9 ± 0.13	0.83 ± 0.08	0.99 ± 0.12	0.01*

A significant difference was ascertained among high elastic modulus, majority of the patients in high elastic modulus were male as compared to female (59.09% vs 26.53%; p=0.001). The LDL cholesterol level (142.12 ± 30.73 vs 127.87 ± 34.64mg/dl; p=0.02) and triglycerides (151.03 ± 15.89 vs 144.89 ± 14.69mg/dl; p=0.03) were significantly higher in high elastic modulus group as compared to low elastic modulus. Further, the cystatin levels were presented with high significant difference when compared between high elastic modulus and low elastic modulus group (0.99 ± 0.12 vs 0.83 ± 0.08mg/l; p=0.01). These findings conclude that the cystatin levels were significantly higher in the high elastic modulus group as compared to the low elastic modulus group. The other biochemical variables were not significant between the groups.

The comparison of other cardiovascular risk factors and history of medication use among the low and high elasticity groups are shown in Table 2. A significant association was manifested among patients with CVD risk factors such as T2DM (p=0.03) and dyslipidemia (p=0.04) and are found to have high elasticity. Patients with statin intake were also observed to be significantly associated with high carotid elasticity (p=0.007).

**Table 2:** Comparison of cardiovascular risk factors and history of medication use among the low and high elasticity groups. * denotes significant p <0.05. OHA: oral hypoglycemic agents

Parameters	Low Eθ (n=63)	High Eθ (n=52)	P value
Type II Diabetes Mellitus			
No	40 (63.5%)	23 (44.2%)	0.03*
Yes	23 (36.5%)	29 (55.8%)	
Systemic hypertension			
No	40 (63.5%)	26 (50%)	0.14
Yes	23 (36.5%)	26 (50%)	
Dyslipidemia			
No	44 (69.8%)	27 (51.9%)	0.04*
Yes	19 (30.2%)	25 (48.1%)	
Smoking			
No	51 (81%)	38 (73.1%)	0.31
Yes	12 (19%)	14 (26.9%)	
Alcohol Intake			
No	54 (85.7%)	37 (71.2%)	0.06
Yes	9 (14.3%)	15 (28.8%)	
Statin Intake			
No	48 (76.2%)	27 (51.9%)	0.007*
Yes	15 (23.8%)	25 (48.1%)	
OHA intake			
No	47 (74.6%)	36 (69.2%)	0.52
Yes	16 (25.6%)	16 (30.8%)	
Anti-hypertensive intake			
No	40 (63.5%)	26 (50%)	0.14
Yes	23 (36.5%)	26 (50%)	

**Figure 1:**
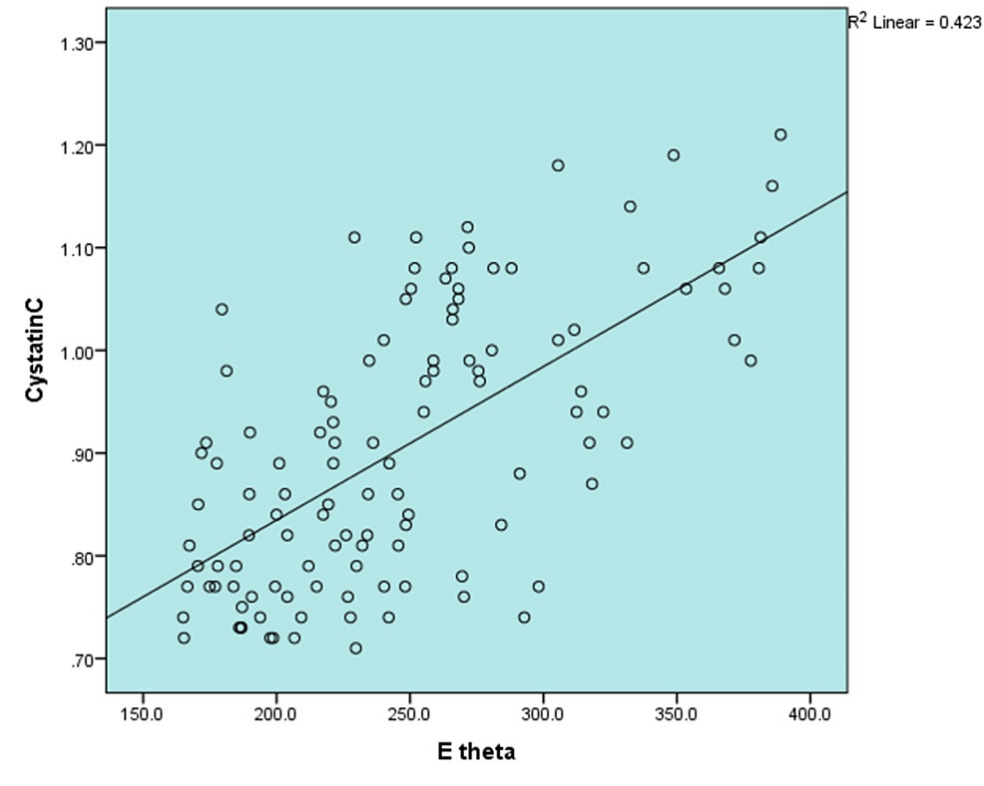
Scatter plot diagram showing correlation between carotid elasticity (kPa) and cystatin.

The associations of carotid elasticity with demographics and cardiovascular risk factors were evaluated and are elaborated in Table 3. Positive correlations with carotid elasticity modulus were observed among variables such as age (p=0.003), SBP (p=0.03), DBP (p=0.005), total cholesterol (p=0.04), LDL (p=0.006), TGL (p=0.01), and cystatin (p<0.001). Meanwhile, scatter plot diagram of carotid elastic modulus and cystatin C level (mg/L) demonstrated a strong correlation of cystatin C with carotid elasticity (r=0.650) (Figure 1).

**Table 3:** Correlation between carotid elasticity and demographics and biochemical parameters. *denotes significant p <0.05. SBP: systolic blood pressure; DBP: diastolic blood pressure; BMI: body mass index; BUN: blood urea nitrogen; FBS: fasting blood sugar; PPBS: post prandial blood sugar; HbA1c %: glycated hemoglobin; LDL: low density lipoprotein; HDL: high density lipoprotein; VLDL: very low density lipoprotein; TGL: triglycerides; IMT: intima-media thickness

Parameters	R value	P value
Age	0.275	0.003
SBP	0.198	0.03*
DBP	0.257	0.005*
HbA1c %	0.122	0.19
Total cholesterol	0.188	0.04*
LDL	0.254	0.006*
HDL	-0.123	0.18
VLDL	-0.123	0.18
TGL	0.229	0.01*
IMT	-0.018	0.85
Cystatin C	0.650	<0.001*

Multiple linear regression illustrates a significant association of cystatin C with carotid elastic modulus (β=0.509; p<0.001). As cystatin C increases carotid elastic modulus also increases. Heart rate (beats/min), albumin and usage of statin were also strongly associated with carotid elastic modulus.

Multiple linear regression analysis of carotid elastic modulus (low and high) and its association with risk factors was evaluated. A significant association was observed between statin users and low carotid elastic modulus (β=0.676; p=0.01). Meanwhile, LDL displayed significant negative association with low carotid elastic modulus (β=-0.355; p=0.04) and cystatin C displayed significant positive association with high carotid elastic modulus (β=0.511; p=0.02).

**Figure 2:**
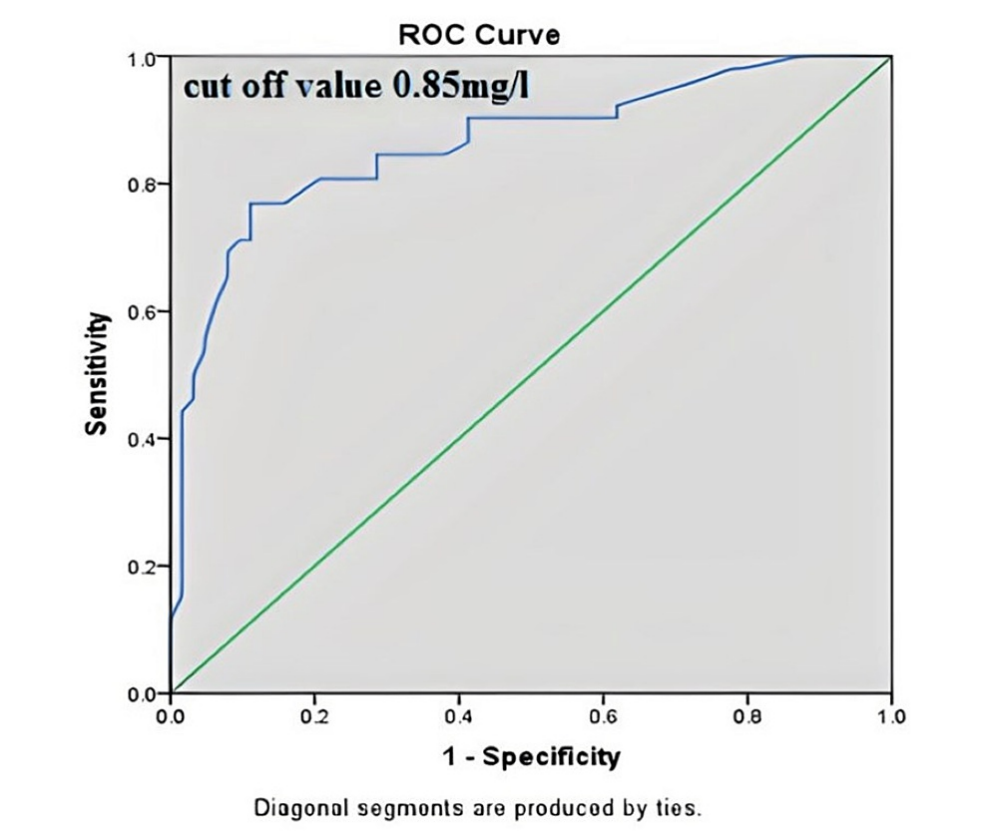
Receiver operating characteristic (ROC) curve analysis of cystatin C (mg/L) in predicting carotid elasticity (high status).

Cystatin C is a better predictor of high carotid elastic modulus with a sensitivity and specificity of around 85%. The cut-off value was measured to be 0.85mg/dl. The area under the curve for predicting early atherosclerosis was 0.864 and it was found to be significant (p<0.001). The ROC curve is shown in Figure 2.

Our study revealed that IMT was only associated with SBP (p=0.027) and DBP (p=0.051). Further, the analysis illustrates the absence of a significant association between cystatin and IMT (r=-0.078; p=0.40).

## Discussion

The present study was carried out to evaluate whether serum cystatin C level is linked with diagnosis of early-stage atherosclerosis. We analyzed the association between subjects with established CVD risk factors and patients with CIMT ≤0.8mm, who are classified as not having atherosclerosis according to the IMT criteria [20-21]. The phased tracking method was assessed to calculate carotid artery elastic modulus.

In our study around 63 patients (54.8%) were observed to have low carotid elastic modulus and 52 patients (45.2%) were observed to have high carotid elastic modulus as similar to the study conducted by Kaneko et al. [22]. Further, in this study LDL-C levels and triglyceride levels are significantly elevated in patients with high carotid elastic modulus compared to the low carotid elastic modulus group, whose observation was also similar to the study conducted by Kaneko et al. In this study, higher proportions of males are in the high carotid elastic modulus category as compared to females, which was similar to the study observation conducted by Ayoola et al. [7]. CIMT was higher in males than females possibly because males are highly prone to various CVD risk factors. This study manifest increased LDL-C levels among patients with high carotid elastic modulus as compared to low carotid elastic modulus group and found to possess significant difference among them (115.0 ± 34.7 vs 102.3 ± 35.5mg/dl; p<0.05). High significant differences were observed between the CVD risk factors and carotid elastic modulus. This finding concludes that high carotid elastic modulus was highly manifested among T2DM patients (p=0.03) and dyslipidemia patients (p=0.04). Meanwhile, patients with statin intake were significantly associated with high carotid elastic modulus (p=0.007) which was relatively similar to the study conducted by van Mi et al. [23]. CIMT level was elevated among patients with T2DM, dyslipidemia and higher statin use patients. 

In our study, the CVD risk factors such as age, systemic hypertension, total cholesterol, LDL, triglycerides and cystatin were positively correlated with carotid elastic resistance (p<0.001). The results of multi-linear regression reveal an association between cystatin C and carotid elasticity. As cystatin C increases carotid elasticity also increases. Albumin and usage of statins are also strongly associated with carotid elasticity. Cystatin C is a better predictor of high carotid elasticity with a sensitivity of around 85%, as similar to the study findings of Kaneko et al. [22] which revealed that carotid elasticity was significantly associated with cystatin C (P<0.01) while IMT was not. In addition, multiple regression analysis elaborates that carotid elasticity was independently associated with cystatin C compared to other variables. Whereas one study done by Zaki et al. [24] showed statistically significant association between cystatin C and IMT (P=0.003).

Carotid arterial IMT is one of the frequently used non-invasive markers for the detection of CVD. Measurement of IMT using USG is a reliable, quick and well-established method. Wide range of studies shows that IMT is useful in the prediction of future myocardial infarction as well as stroke [25-26]. Meanwhile, IMT prediction of early-stage atherosclerosis is not possible, since atherosclerosis can be diagnosed only after the evolution of arterial wall thickening. Indeed, the significance of carotid IMT remains a matter of debate with conventional coronary risk factors predicting future cardiovascular events. In the present study, we enrolled patients with CVD risk factors with IMT less than 0.8mm, who are classified as not having atherosclerosis according to the IMT criteria.

Cystatin C may provide a more sensitive and accurate estimation of renal function than eGFR, as cystatin C is less influenced by age and muscle mass [13]. In this study, we found that serum cystatin C level displayed strong association with carotid elastic modulus which is a reliable marker for early-stage atherosclerosis, while it failed with IMT. Furthermore, multiple regression analysis revealed a strong relationship between cystatin C and Eθ, especially in the subgroup with relatively high carotid elastic modulus. One possible explanation of these results is that renal impairment is associated with atherosclerosis development which can be observed at the initial stage of both disorders. On the other hand, it is also possible that cystatin C might itself be directly involved in the development of atherosclerosis [27]. Cystatin C is an endogenous inhibitor of cysteine protease. Atherosclerosis is an inflammatory disease characterized by remodeling of the extracellular matrix of the arterial walls. Cysteine protease induces degradation of the extracellular matrix. The imbalance of cystatin C results in increased degradation of extracellular matrix and migration of monocytes/macrophages into the intima, thus leading to the development of atherosclerosis. Tissue cystatin C levels are reportedly reduced in atherosclerotic plaques [28-29]. In contrast, inflammatory cytokines stimulate cells outside of the vascular walls to secrete cystatin C into the circulation. Thus, serum cystatin C is considered to be compensatorily up-regulated in subjects who have developed atherosclerosis [30].

This study has some limitations. Despite using a sufficient sample size, only one study center was used for this investigation. As a result, future studies with a larger sample size can be a source to plug in to the significance of potential markers in early detection of atherosclerosis.

## Conclusions

Our current study concludes a significant association between cystatin C levels and calculated arterial wall elastic modulus with 85% of sensitivity and specificity. Hence, cystatin C levels can be used as a reliable biomarker for early detection of atherosclerosis. In patients with one or more of these risk factors, cystatin C level estimation may be explored for early detection of atherosclerosis as a biochemical marker.
